# Impaired Adult Neurogenesis in the Dentate Gyrus of a Triple Transgenic Mouse Model of Alzheimer's Disease

**DOI:** 10.1371/journal.pone.0002935

**Published:** 2008-08-13

**Authors:** José J. Rodríguez, Victoria C. Jones, Masashi Tabuchi, Stuart M. Allan, Elysse M. Knight, Frank M. LaFerla, Salvatore Oddo, Alexei Verkhratsky

**Affiliations:** 1 Faculty of Life Sciences, The University of Manchester, Manchester, United Kingdom; 2 Department of Neurobiology and Behaviour, University of California Irvine, Irvine, California, United States of America; 3 Institute of Experimental Medicine, ASCR, Prague, Czech Republic; Baylor College of Medicine, United States of America

## Abstract

It has become generally accepted that new neurones are added and integrated mainly in two areas of the mammalian CNS, the subventricular zone and the subgranular zone (SGZ) of the dentate gyrus (DG) of the hippocampus, which is of central importance in learning and memory. The newly generated cells display neuronal morphology, are able to generate action potentials and receive functional synaptic inputs, i.e. their properties are similar to those found in mature neurones. Alzheimer's disease (AD) is the primary and widespread cause of dementia and is an age-related, progressive and irreversible neurodegenerative disease that deteriorates cognitive functions. Here, we have used male and female triple transgenic mice (3xTg-AD) harbouring three mutant genes (β-amyloid precursor protein, presenilin-1 and tau) and their respective non-transgenic (non-Tg) controls at 2, 3, 4, 6, 9 and 12 months of age to establish the link between AD and neurogenesis. Using immunohistochemistry we determined the area density of proliferating cells within the SGZ of the DG, measured by the presence of phosphorylated Histone H3 (HH3), and their possible co-localisation with GFAP to exclude a glial phenotype. Less than 1% of the HH3 labeled cells co-localised with GFAP. Both non-Tg and 3xTg-AD showed an age-dependent decrease in neurogenesis. However, male 3xTg-AD mice demonstrated a further reduction in the production of new neurones from 9 months of age (73% decrease) and a complete depletion at 12 months, when compared to controls. In addition, female 3xTg-AD mice showed an earlier but equivalent decrease in neurogenesis at 4 months (reduction of 63%) with an almost inexistent rate at 12 months (88% decrease) compared to controls. This reduction in neurogenesis was directly associated with the presence of β-amyloid plaques and an increase in the number of β-amyloid containing neurones in the hippocampus; which in the case of 3xgTg females was directly correlated. These results suggest that 3xTg-AD mice have an impaired ability to generate new neurones in the DG of the hippocampus, the severity of which increases with age and might be directly associated with the known cognitive impairment observed from 6 months of age onwards . The earlier reduction of neurogenesis in females, from 4 months, is in agreement with the higher prevalence of AD in women than in men. Thus it is conceivable to speculate that a recovery in neurogenesis rates in AD could help to rescue cognitive impairment.

## Introduction

The classical view that all neurones are generated (via neurogenesis) during prenatal development and early postnatal life has been challenged by the seminal study of Altman and Das (1965) [1] and now it is generally accepted that neurogenesis does also occur in adulthood mainly in two areas of the mammalian CNS [1]–[5]. These areas, which are involved in both plasticity and stability of the brain, are the anterior part of the subventricular zone (SVZ) along the lateral ventricles, which is also an important a site of gliogenesis [6], [7] and the subgranular zone (SGZ) of the dentate gyrus (DG) of the hippocampus [4], [5]. In both areas neurogenesis progress as a complex multi-step process which starts with the proliferation of precursors residing in the SVZ or in the SGZ. For the hippocampus, it has been estimated that several thousand new cells are generated daily [5]. However, within several days after their birth at least fifty percent of the newborn cells die [5]. The cells surviving this initial period of cell death differentiate mainly into granule neurones and endure for several months. These newly generated neurones receive synaptic inputs, extend axons along the mossy fibres tract and exhibit electrophysiological properties similar to those of mature dentate granule cells [8], [9]. In addition these new cells express a full complement of membrane receptors [10]. From a functional point of view, hippocampal neurogenesis plays an important role in memory processes. Decline in neurogenesis within SGZ has been involved in cognitive impairments linked with ageing and neurodegenerative disorders, and was suggested to play a role in Alzheimer disease (AD) [5], [11].

AD is a progressive neurodegenerative disease which is the primary cause of dementia in the elderly and is characterized by damage of the brain regions associated with learning and memory, such as the hippocampus [12], [13]. Decline in neurogenic capacity could participate in AD-associated cognitive impairments and contribute to early AD symptoms such as the inability to acquire and store new information [14]–[16]. Incidentally, the use of endogenous neuronal precursors to replace lost and/or damaged cells has been proposed as a potential therapeutic approach to treat AD [17], [18]. Experimental studies of neurogenesis in various AD animal models, however, resulted in contradictory findings [19]–[26]. For example, animals carrying presenilin or some APP mutant genes demonstrated an impaired neurogenesis in the DG [20]–[22], [24], [27], [28]. Conversely, recent studies, performed on the APP_Sw, Ind_ (Swiss/Indian mutation) PDGF-APP mutant and on FAD post-mortem human material [18], [29], reported an increase of neurogenesis. It has to be noted, though, that the study on human FAD cases analysed only immature newly generated neurones without providing definitive probes of further development and/or progress into mature cells.

In the present study we sought to determine the rate and possible changes in hippocampal neurogenesis in the recently developed triple-transgenic AD (3xTg-AD) mouse model, that harbours the mutant genes for amyloid precursor protein (APP_Swe_), for presenilin 1PS1_M146V_ and for tau_P301L_ [30], [31]. These animals are recognised as relevant AD model since they show temporal- and region-specific Aβ and tau pathology, which closely resembles that seen in the human AD brain [30], [31]. As well as progressively developing plaques and tangles the 3xTg-AD mice also show clear functional and cognitive impairments including LTP, spatial memory and long term memory deficits; which are manifest in an age-related manner importantly preceding the appearance of histological markers [30], [31]. Cognitive deficits in the 3xTg-AD model correlate with the accumulation of intraneuronal Aβ [30]–[34]. Subsequently, we decided to investigate a gender difference in the number of proliferating cells, because it is well establish that AD affects women more than men [35], [36]. Finally we also aimed to correlate the rate of neurogenesis with the presence of intracellular β-amyloid.

## Materials and Methods

### Mice

All animal procedures were performed according to the Animal Scientific Procedures Act of 1986 under the license from the United Kingdom Home Office.

The generation of the 3xTg-AD mice was done as previously described [30], [31], [37]. Briefly, human amyloid precursor protein with the Swedish mutation (APP_Swe_) and human tau with the P301L (tau_P301L_) mutation were microinjected into single-cell embryos from homozygous presenilin 1PS1_M146V_ knockin mice. The background of the PS1 knockin mouse is a hybrid 129/C57BL6. The Non-Tg mice used were from the same strain and genetic background as the PS1 knockin mice, but they harbor the endogenous wild-type mouse PS1. All 3xTg-AD and Non-Tg mice were obtained from crossing homozygous breeders. Male and female mice were independently group housed and kept on daily 12 h light-dark cycles dark schedule. All mice were given ad libitum access to food and water.

### Fixation and tissue processing

Male and female 3xTg-AD and their respective non-transgenic (non-Tg) controls were anaesthetized with an intraperitoneal injection of sodium pentobarbital at different time points (2, 3, 4, 6, 9 and 12 months of age; n = 3–7). The brains were fixed by perfusion through the aortic arch with 25 ml of 3.8% acrolein (TAAB, UK) in a solution of 2% paraformaldehyde and 0.1 M phosphate buffer (PB) pH 7.4, followed by 75 ml of 2% paraformaldehyde. Brains were removed from the cranium and cut into 4–5 mm coronal slabs of tissue containing the entire rostrocaudal extent of the hippocampus. This tissue was then post-fixed for 30 minutes in 2% paraformaldehyde and sectioned at 40–50 µm on a vibrating microtome (VT1000, Leica, Milton Keynes, UK). To remove excess reactive aldehyde groups, sections were treated with 1% sodium borohydride in 0.1 M PB for 30 minutes. The tissue sections were then freeze-thawed to optimize the penetration of immunoreagents. For this procedure, sections were incubated in cryoprotectant solution containing 25% sucrose and 3.5% glycerol in 0.05 M PB at pH 7.4 and subsequently rapidly immersed in chlorodifluoromethane followed by liquid nitrogen and then thawed at room temperature in PB. Sections were then rinsed in 0.1 M PB followed by 0.1 M Tris-buffered saline (TBS), pH 7.6.

### Antibodies

A polyclonal affinity-purified rabbit antiserum raised against phosphorylated Histone 3 (Upstate, USA; #06-570) and a monoclonal mouse antiserum generated against GFAP from pig spinal cord (Sigma-Aldrich Company Ltd., UK; #G3893) were used for the determination of proliferating cells and glia. Specificity of these antisera was confirmed by immunoblot and western blot [38]. For identification of intracellular beta amyloid (Aβ) deposits we used a monoclonal mouse antiserum that reacts with abnormally processed isoforms, as well as precursor forms of Aβ, recognizing an epitope within amino acids 3–8 (EFRHDS; anti-Ab 6E10 [SIG-39320]. Signet Laboratories, Dedham, MA).The immunolabelling pattern we obtained with this antibody is equivalent to that obtained previously in different brain regions [30], [31].

To assess for non-specific background labelling or cross reactivity between antibodies derived from different host species, a series of control experiments were performed. Omission of primary and/or secondary antibodies from the incubation solutions resulted in a total absence of target labelling. These primary antibodies are therefore regarded as specific to their designated targets.

### Immunohistochemistry

To optimize detection of all HH3 and GFAP cells and containing profiles we used the highly sensitive avidin–biotin peroxidase complex (ABC) method [39]; and to minimize methodological variability, sections through the dorsal hippocampus containing both hemispheres of all animals were processed at the same time using precisely the same experimental conditions. For this procedure, the vibratome sections were first incubated for 30 minutes in 0.5% bovine serum albumin in TBS to minimize non-specific labelling. The tissue sections were then incubated for 48 hours at 4°C in 0.1% bovine serum albumin in TBS containing: (1) rabbit polyclonal antiserum for HH3 (1∶1,000) and (2) mouse monoclonal antiserum for GFAP (1∶60,000). Subsequently, the HH3 and GFAP antibodies were detected in a sequential manner on the same sections. For HH3 labelling, sections were washed and placed in (1) 1∶200 dilutions of biotinylated donkey anti-rabbit IgG (Jackson Immunoresearch, Stratech Scientific Ltd., Soham, UK) and (2) 1∶200 dilutions of biotin-avidin complex from the Elite kit (Vector Laboratories Ltd., Peterborough, UK). All antisera dilutions were prepared in TBS, and the incubations were carried out at room temperature. The peroxidase reaction product was visualized by incubation in a solution containing 0.022% of 3,3′ diaminobenzidine (DAB, Aldrich, Gillingham, UK) and 0.003% H_2_O_2_ in TBS for 6 minutes. For GFAP labelling, sections were then rinsed again in TBS and incubated (1) 1∶200 dilution of biotynilated horse anti-mouse IgG (1∶200; Vector Laboratories Ltd., Peterborough, UK) and (2) placed in a 1∶200 dilution of biotin-avidin complex from the Elite kit (Vector Laboratories Ltd., Peterborough, UK). The GFAP peroxidase product was then visualized in a solution prepared from the Novared or SGZ kits (Vector Laboratories Ltd., Peterborough, UK) for 3–4 minutes. This allowed us to see the GFAP labelling in red and/or blue respectively; allowing us to differentiate it from the HH3 labelled cells (brown).

The same immunoperoxidase approach, but for single labelling, was used for the detection of intracellular Aβ. Briefly, adjacent sections were incubated for 48 hours at 4°C in 0.1% bovine serum albumin in TBS containing mouse monoclonal antiserum for Aβ (1∶2000). Subsequently, sections were then washed and placed in (1) 1∶200 horse anti-mouse IgG (Vector Laboratories Ltd., Peterborough, UK) and (2) 1∶200 dilution of biotin-avidin complex from the Elite kit (Vector Laboratories Ltd., Peterborough, UK). The peroxidase reaction product was visualized by incubation in a solution containing 0.022% of 3,3′ diaminobenzidine (DAB, Aldrich, Gillingham, UK) and 0.003% H_2_O_2_ in TBS for 6 minutes.

### HH3 Area density and Aβ cell number

To determine the area density (Sv, number/mm^2^) of HH3-immunoreactive neurons, the labelled cells were counted on both hemispheres in six non-consecutive coronal Vibratome sections, separate by at least 80 µm, taken through representative sections of both the dorsal (3) and ventral (3) DG of the hippocampus at levels 1.22 mm/2.46 mm and 2.54 mm/3.80 mm posterior anterior to bregma, respectively, according to the mouse brain atlas of Paxinos and Watson (1986) [40]. The number of HH3 positive cells and the area measurements of the complete dentate gyrus and its different layers (granule cell layer –GCL-, molecular layer –ML- and hilus) were determined blindly.

The number of Aβ containing neurones was examined in the CA1 region of the hippocampus CA1, since this field shows the earliest and strongest accumulation of Aβ intracellular deposits. This quantification was carried out on six non-consecutive hippocampal sections of the same animals used for the proliferation analysis.

### Statistical analysis

An analysis of variance (ANOVA) was used to examine differences in the mean area density of labelled HH3 cells between the 3xTg-AD and non-Tg animals and sexes, followed by unpaired *t*-test comparisons at the different time points. Spearman correlation was used to correlate the mean area density of HH3 positive cells with the mean number of Aβ containing neurons (implemented through GraphPad Prism 4.0, GraphPad Software, Inc.).

## Results

**Figure 1:**
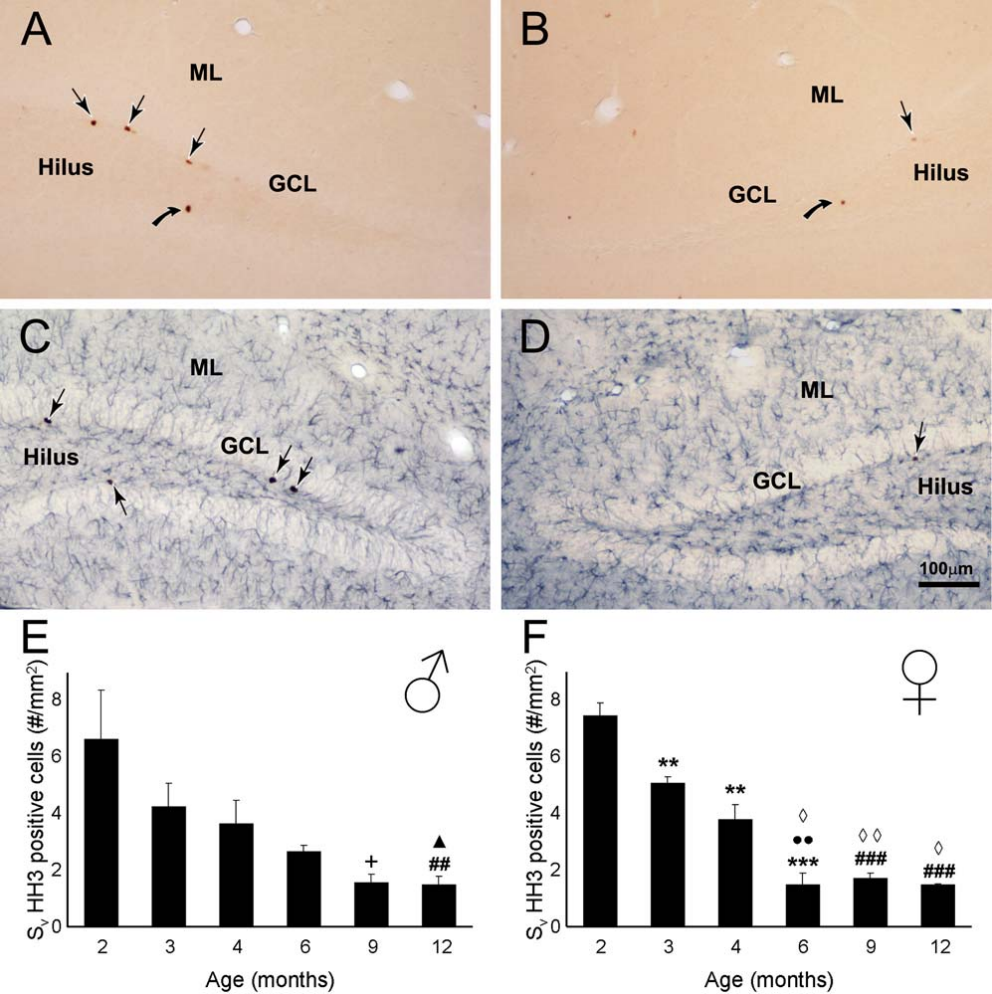
Photomicrographs showing phosphorilated Histone H3 (HH3, a proliferating mitotic marker) within the dentate gyrus of Non Tg mice. A–B: Single labelling of HH3 positive cells (arrows) in the dentate gyrus of 2 (A) and 12 months (B) Non Tg mice. C–D: Dual labeling of HH3 positive cells (arrows) and glial cells (GFAP, blue) in the dentate gyrus of 2 (C) and 12 months (D) Non Tg mice. E–F: Bar graphs showing the area density of HH3 positive cells within the dorsal dentate gyrus (all layers included) of Non-Tg males (E) and females (F) mice. GCL: Granular Cell Layer, ML: Molecular Layer. ** = p<0.01 compared to 2 months; *** = p<0.001 compared to 2 months;^••^ = p<0.01 compared to 3 months; ## = p<0.01 compared to 2 and 3 months; ### = p<0.001 compared to 3 months; ◊ = p<0.05 compared to 4 months; ◊◊ = p<0.01 compared to 4 months; ∧ = p<0.05 compared to 4 and 6 months; + = p<0.05 compared to 2, 3 and 6 months.

**Figure 2:**
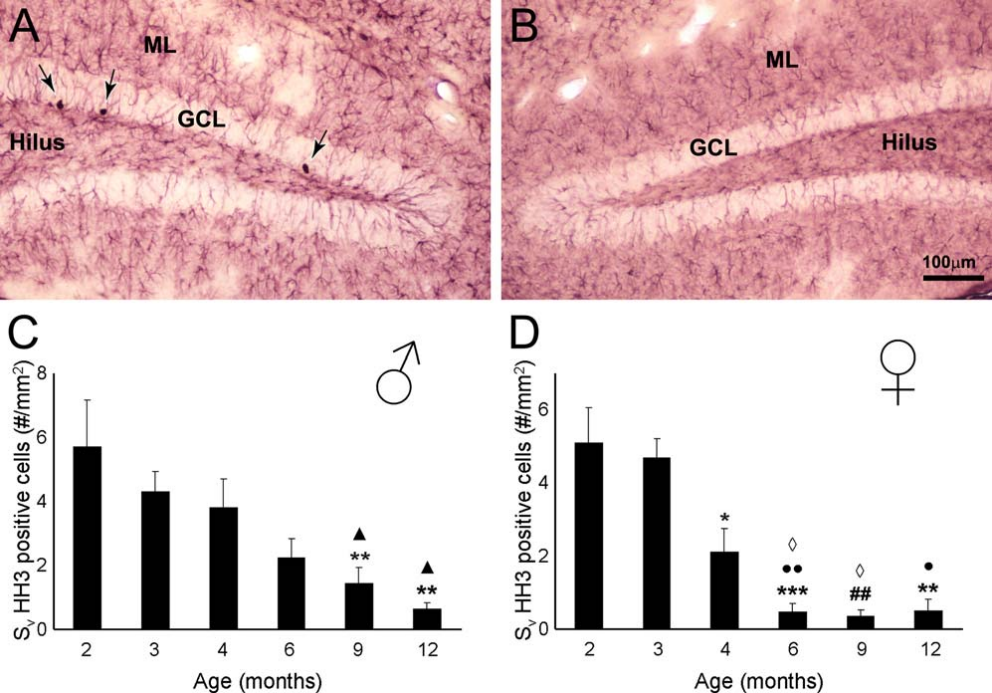
Brightfield micrographs showing HH3 labelled cells within the dentate gyrus of 3xTg-AD e. A–B: Dual labeling of HH3 positive cells (arrows) and glial cells (GFAP, red) in the dentate gyrus of 2 (A) and 12 months (B) 3xTg-AD mice. C–D: Bar graphs showing the area density of HH3 positive cells within the dorsal dentate gyrus (all layers included) of 3xTg-AD males (C) and females (D) mice. GCL: Granular Cell Layer, ML: Molecular Layer. ** = p<0.01 compared to 3 months; *** = p<0.001 compared to 3 months; ^•^ = p<0.05 compared to 2 months ^••^ = p<0.001 compared to 2 months; ## = p<0.01 compared to 2 and 3 months; ◊ = p<0.05 compared to 4 months; ∧ = p<0.05 compared to 2 and 4 months.

In the dorsal hippocampus and more specifically within the dentate gyrus of both non-Tg and 3xTg-AD mice a fair number of newly generated cells could be visualized, as indicated by HH3 immunoreactivity (HH3-IR; Figs.1A, 2A). These newly formed cells showed the distinctive characteristics of proliferating cells; they were mainly localized in the inferior part of the granule cell layer (GCL) and demonstrated typical morphology such as irregular shape and small size; sometimes they appeared close together and/or formed clusters (Fig. 1A–B).

### Effects of ageing on neurogenesis in non-Tg animals

**Figure 3:**
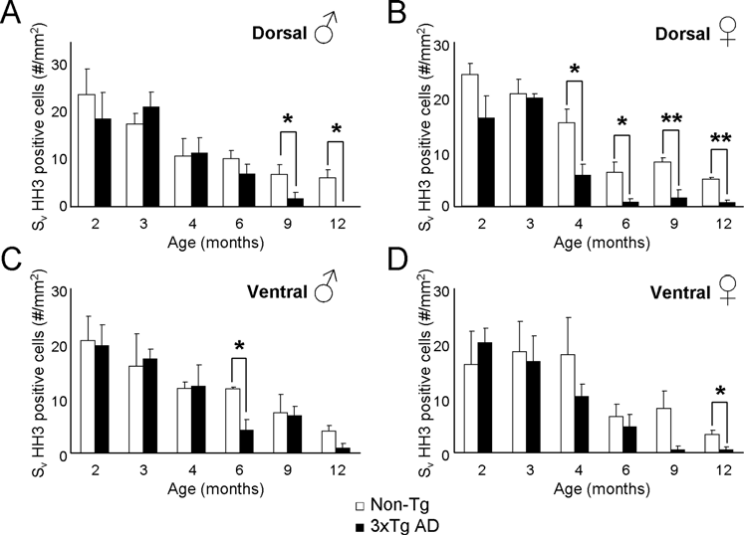
Bar graphs showing the mean area density HH3 labelled cells within the GCL of the dentate gyrus of both 3xTg-AD and control nonTg-AD mice. A–B: Males (A) and females (B) dorsal GCL. C–D: Males (C) and females (D) dorsal GCL. Asterisks indicate a significant difference in the means.

Quantitative analysis of the rate of cell proliferation showed a reduction in the number of HH3-IR cells with age. This reduction was apparent within the dentate gyrus of non-Tg males and females mice (F_5,20_ = 5.643, p = 0.0021 and F_5,16_ = 44.8, p<0.0001, respectively; Fig 1E–F) and in the GCL where we observed the highest number of proliferating cells (F_5,20_ = 5.362, p = 0.0028 and F_5,16_ = 17.54, p<0.0001, respectively; Fig 3A–B). Decrease in proliferation rate with age was quite significant: at 6 months of age in both sexes the proliferation rate was reduced by more than 60% when compared to the 2 months old animals (males 2.64±0.23 vs. 6.61±1.74; females 1.48±0.39 vs. 7.46±0.45, Fig. 1E–H). Decrease in the number of HH3 cells in dentate gyrus and the subgranular zone of the GCL appears earlier in females (3–4 month) than in males as confirmed by the *t*-test analysis (Figs. 1E–F; Fig. 3).

Dual labelling showed that the majority of HH3-IR cells did not possess the astroglia marker GFAP; in fact, less then 1% of HH3-IR cells co-expressed GFAP in the dentate gyrus, including the GCL (data not shown).

### Impairment of neurogenesis in 3xTg-mice

Similarly to the controls, the rate of neurogenesis in 3xTg-AD mice was decreased with age (Fig. 2, 3) in both males and females throughout the dentate gyrus (F_5,20_ = 5.437, p = 0.0026 and F_5,18_ = 12.39, p<0.0001, respectively) and in the GCL (F_5,20_ = 8.524, p = 0.0002 and F_5,18_ = 11.81, p<0.0001, respectively; Figs 2C–D). Decrease in proliferation rate in 3xTg-AD animals was about 60–90% more pronounced compared to control animals: at 6 months compared to the 2 months 3xTg-AD mice (males 2.23±0.5 vs. 5.71±1.47; females 0.47+0.23 vs 5.09±1) Fig. 2 C–D). Furthermore, at 12 months of age males and females showed very little capacity of forming new cells within the GCL (Fig. 2 C–D; Fig. 3); whilst in both males and females non-Tg animals we could still observe approximately a 20–35% of the number of HH3-IR observed at young ages (2–3 months ; Fig 3).

### Neurogenesis depression in 3xTg-mice is gender dependent

When we analysed the number of HH3-IR cells within the GCL of the 3xTg-AD mice and compared it with the cell proliferation rate of the non-Tg it became evident that at young ages (2 and 3 months) the levels were very similar whilst at older ages, especially 9 and 12 months the neurogenesis levels have decreased over 70% in both groups (Figs. 1 E–F, 2 C–D, 3). The quantification and consequent statistical analysis showed that the age associated reduction in HH3-IR cells in 3xTg-AD males compared to non-Tg animals start to be significant at 9 months (73%; showing a trend to significant difference) and is completely disappeared at 12 months of age (Fig. 3A; age effect, F_5,1_ = 53.57, p<0.0001; group effect F_5,1_ = 1.73, p = 0.1744; age x group effect F_5,1_ = 3.75, p = 0.5353; p<0.05 at 12 months). In contrast in females 3xTg-AD, compared to non-Tg animals, HH3-IR cells were already significantly reduced at 4 months of age (63%) being maximal (88% reduction) at 12 months (Fig. 3B; age effect, F_5,1_ = 68.08, p<0.0001; group effect F_5,1_ = 10.31, p<0.0001; age x group effect F_5,1_ = 2.31, p = 0.4961; p<0.05 at 4 and 6 months; p<0.001 at 9 and 12 months). This impairment in neurogenesis rate within the 3xTg-AD mice is mainly due to changes at dorsal more than ventral hippocampal levels (Fig. 3), which is consistent with their specific preferential roles in learning and memory and affective behaviour respectively [41].

### Relationship between intraneuronal β-amyloid accumulation and neurogenesis

**Figure 4:**
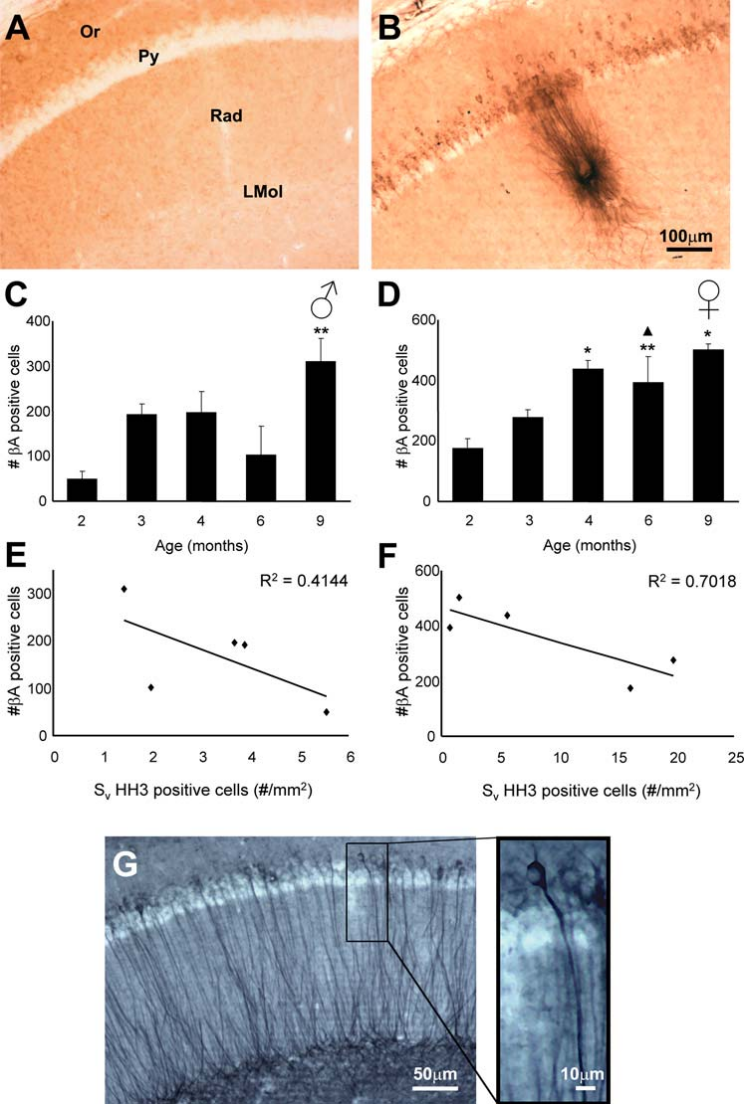
Photomicrographs showing the presence of β-amyloid within the pyramidal neurones of CA1 as well as the presence of a plaque in 12 months 3xTg-AD mice (B) compared to a nonTg control animal (A). C–D: Bar graphs showing the number of cells containing β-amyloidin the hippocampal CA1 of males (C) and females (D) 3xTg-AD mice. E–F: Linear correlations between the mean number of cells containing β-amyloid in the hippocampal CA1 and the mean area density of HH3 positive cells in the GCL of the dentate gyrus of males (E) and females (F) 3xTg-AD mice. In G we can see the accumulation phosphorilated Tau within the CA1 of a 3xTg-AD mice. Or: CA1 Stratum Oriens, Py: CA1 stratum Pyramidale, Rad: CA1 Stratum Radiatum, LMol: CA1 Stratum Lacunosum Moleculare. * = p<0.05 compared to 2 months; ** = p<0.01 compared to 2 months; ∧ = p<0.05 compared to 3 months.

The 3xTg-AD mice had also an age-dependent increase in the number of hippocampal neurones accumulating β-amyloid (Fig. 4A–D). Within the hippocampus neurones containing β-amyloid could be found as early as 2 months of age, and this number was higher in females compared to males (Fig. 4C–D). Quantitative analysis showed that in both males and females 3xTg-Ad mice there was an age-dependent increase of β-amyloid containing neurones that was maximal and very significant at 9 months of age when compared to young animals (Fig. 4 C–D; F_4,10_ = 4.231, p = 0.0293 and F_4,10_ = 4.948, p = 0.0184, respectively). The fully formed β-amyloid plaques within the neuropil, however, were observed much later, at 9 and 12 months (Fig. 4B). Increase in the number of β-amyloid containing hippocampal neurones seem to match with the reduction of GCL proliferating cells in both males and females (Fig. 4E–F). However, only females showed a significant indirect correlation between the number of HH3-IR cells with the number of β-amyloid positive cells (R^2^ = 0.7018; Fig 4F). This finding showing a higher prevalence of β-amyloid positive cells in 3xTg-AD females is also in agreement with the recently reported 20% increase in plaque load observed in APP23 transgenic mice females when compared to males [42]. Furthermore,The presence of high number of β-amyloid containing neurones and occurrence of plaques at 12 months were occasionally concomitant with the first appearance of phosphorilated Tau (Fig. 4G).

## Discussion

In the present study we have used single and dual labelling immunohistochemistry to demonstrate changes in cell proliferation and neurogenesis within the hippocampal dentate gyrus of an AD animal model. The experiments were performed on a recently developed 3xTg-AD mouse model, which is recognised as an extremely relevant, since these transgenic animals show temporal- and region-specific Aβ and tau pathology, which closely resembles that seen in the human AD brain [30], [31]. These pathological hallmarks are concomitant with clear functional and cognitive impairments including LTP, spatial memory and long term memory deficits, which are manifest in an age-related manner [30]–[34]. We found that only a very small proportion of HH3-IR proliferating cells in either 3xTg-AD or Non-Tg mice express GFAP (<1%), suggesting that those HH3-IR cells are likely of a neuronal lineage and thus are an indicator of neurogenesis.

Our main findings are that 3xTg-AD mice have a decreased GCL neurogenesis, which is also is gender dependent. As happens in normal rodents (including our control non-Tg animals) the decrease in the dentate gyrus GCL neurogenesis develops with age [5], [14] However, we found that this effect is much exacerbated in 3xTg-AD, being at least 60% stronger than in normal animals. These results are in agreement with findings in other transgenic models of AD in which transgenic mice having mutant forms of APP or presenilin-1 demonstrated impaired neurogenesis [20]–[22], [24], [27], [28]. Since none of models used previously fully reproduces the features of familial and/or sporadic AD, our findings made in the 3xTg-AD mice become of major relevance and importance.

We also demonstrated that the impairment of neurogenesis in the dentate gyrus of the hippocampus is closely associated with AD pathogenesis. Indeed hippocampus is affected early in AD; impaired memory related to hippocampal damage may be associated with deregulations of neurogenesis [5], [11], [29], [43]. Two previous studies, however, are in contradiction with our findings; one performed on the APP_Sw, Ind_ (Swiss/Indian mutation) mutant and one on FAD post-mortem human material, reporting an increase of neurogenesis [18], [29]. These discrepancies could be explained by methodological and preservation differences, including the post-mortem fixation delay which could contribute to antigen masking and in consequence a misevaluation of the proliferation rates [44]. In addition, in these studies the Doublecortin (Dcx) labelling, which marks both young and immature neurons was used. This could affect the data interpretation because more than 50% of the newborn cells die [5]. Another discrepancy may reside in the fact that even if they use 5-Bromo-2′-Deoxyuridine (BrdU) as an accurate index to label proliferating cells, very few of them survives to 4 weeks [28]. Furthermore, these methods suffer from uncertainty since they may not detect a distinct proliferative state but instead mark repaired DNA in post-mitotic neurons and/or an abortive cell cycle [45], [46].

In this study we also investigated the gender difference of the rate of neurogenesis in 3xTg-AD mice. We found that neurogenesis in female 3xTg-AD mice is affected earlier; already at 4 months of age we found significant depression of neurogenesis in female AD animals. This difference is in line with the recently reported sexual dimorphism observed in cognitive performance such as the Morris water-maze in which female 3xTg-AD mice also perform worse than males [37]. In addition, it correlates to the well known fact, that AD affects women earlier and with more severity than men (according to some findings the incidence of the disease is double in females [35], [36]. Importantly such a gender-based predisposition toward females is specific of AD and not found in other dementias [36]. Several lines of evidence suggest that this prevalence is directly related with the circulating levels of estrogens [35], [36], [47]. As a result females have higher levels of cell proliferation, but not cell survival when compared with males cell proliferation rate in turn depends on the endocrine status [48]. Only proestrus females, with high levels of estradiol, show higher levels of cell proliferation; which seems to be mediated through its effect on estrogen receptors [34], [48], [49]. Be it all as it may, all these results are in agreement with our findings of a greater degree of Aβ pathology in female versus male 3xTg-AD animals from 4 months of age; this is also in agreement with recent results observed in anothere AD transgenic mouse model (APP23) as well as in line with clinical evidence of higher prevalence of AD in females [35], [36], [42], [50], [51]. However, future studies are needed to confirm this estrogen active role on cell proliferation and neurogenesis whilst lately it has been shown controversial evidence in some mouse strains, such as C57BL/6, in which female hippocampal cell proliferation is not influenced by estrous cycle or ovariectomy [52].

Generation of new neurones is an important feature of the adult brain; in hippocampus the newborn neuronal cells, governed by multiple factors, undergo complex stages of morphological and functional maturation and integrate into existing neural circuitry [53]. The hippocampal neurogenesis can be directly involved in variety of cognitive processes; decreased neurogenesis negatively affects certain learning and memory processes such as spatial memory [51], [53]. In contrast, increased load on the cognitive processes (e.g. enriched environment) and physical exercises positively affect neurogenesis [54]. On the other hand the role of impaired neurogenesis in cognitive deficits in neurodegenerative diseases is much less characterized. However, our observations showing an impairment in neurogenesis correlate with the recent evidence of age dependent impairment in spatial and long-term memory tasks observed in the 3xTg-AD animals [32]–[34]; suggesting a critical implication of neurogenesis in cognitive deficits

Thus, we consider that our data are specifically important as we directly addressed the contradicting issue of the neurogenesis status in the AD. By using longitudinal study on newly developed transgenic model of the disease we demonstrated clear inhibition of neurogenesis in diseased animals; this inhibition is specifically pronounced in females, which correlates with clinical observations. Therefore we conclude that inhibited neurogenesis can play an active role in development of cognitive deficits and progression of the AD.
